# Optimisation of a Greener-Approach for the Synthesis of Cyclodextrin-Based Nanosponges for the Solubility Enhancement of Domperidone, a BCS Class II Drug

**DOI:** 10.3390/ph16040567

**Published:** 2023-04-10

**Authors:** Mohit Vij, Neha Dand, Lalit Kumar, Pankaj Wadhwa, Shahid Ud Din Wani, Wael A. Mahdi, Sultan Alshehri, Prawez Alam, Faiyaz Shakeel

**Affiliations:** 1 School of Pharmaceutical Sciences, Lovely Professional University, Phagwara 144411, India; 2 Government Pharmacy College, Kangra Nagrota Bagwan, Matyari 176047, India; 3 Department of Pharmaceutics, Bharati Vidyapeeth’s College of Pharmacy, Navi Mumbai 400614, India; 4 Sri Sai College of Pharmacy, Amritsar 143149, India; 5 Department of Pharmaceutical Sciences, School of Applied Science and Technology, University of Kashmir, Srinagar 190006, India; 6 Department of Pharmaceutics, College of Pharmacy, King Saud University, Riyadh 11451, Saudi Arabia; 7 Department of Pharmaceutical Sciences, College of Pharmacy, AlMaarefa University, Ad Diriyah 13713, Saudi Arabia; 8 Department of Pharmacognosy, College of Pharmacy, Prince Sattam bin Abdulaziz University, Al-Kharj 11942, Saudi Arabia

**Keywords:** domperidone, cyclodextrin, nanosponge, solubility, bioavailability, microwave-assisted synthesis

## Abstract

BCS class II molecules suffer from low oral bioavailability because of their poor permeability and sub-optimal aqueous solubility. One of the approaches to enhance their bioavailability is using cyclodextrin-based nanosponges. This study aimed to optimise and evaluate the feasibility of a microwave-assisted approach to synthesise nanosponges and improve domperidone’s solubility and drug delivery potential. In the production process, microwave power level, response speed, and stirring speed were optimised using the Box-Behnken approach. Ultimately, the batch with the smallest particle size and highest yield was chosen. The optimised method of synthesis of the nanosponges resulted in a product yield of 77.4% and a particle size of 195.68 ± 2.16 nm. The nanocarriers had a drug entrapment capacity of 84 ± 4.2% and a zeta potential of −9.17± 0.43 mV. The similarity and the difference factors demonstrated proof-of-concept, showing that the drug release from the loaded nanosponges is significantly greater than the plain drug. Additionally, spectral and thermal characterisations, such as FTIR, DSC, and XRD, confirmed the entrapment of the drug within the nanocarrier. SEM scans revealed the porous nature of the nanocarriers. Microwave-assisted synthesis could be used as a better and greener approach to synthesise these nanocarriers. It could then be utilised to load drugs and improve their solubility, as seen in the case of domperidone.

## 1. Introduction

A medicine with a Biopharmaceutical Classification System (BCS), class II or IV classification, has poor aqueous solubility, which may limit oral absorption and result in subpar oral bioavailability [1]. Drug candidates whose advancement is hampered by insufficient solvency and slow disintegration have been subjected to several tactics to improve their attractiveness. Structure-based and formulation-based methodologies can be applied to improve a drug’s dissolvability and disintegration rates. The focus here is on cyclodextrin-based nanosponges (CDNS) as potential nanocarriers for enhancing the target drug domperidone (DOM) solubility. DOM, a BCS class II drug that is insufficiently water-soluble, is a selective blocker of D2 and D3 dopamine receptors present in the chemoreceptor trigger zone and is utilised as an antiemetic and stomach prokinetic agent [2].

Natural and inorganic nanosponges are prospective classes of nanocarriers that can address the pharmacokinetic challenges faced by poorly soluble drugs [3]. Solid particles with cross-sectional holes on the surface and nanosized pits on the inside make up three-dimensional sponge-like nanosponges, which can carry a range of medications, from small synthesised compounds to large proteins and peptides [4]. Both lipophilic and hydrophilic actives can be conveyed through these nanosponges. Because of their focus on increasing solubility and designing customised delivery mechanisms, nanosponges may transform the treatment of many diseases [5].

Depending on the type of polymer used, there are many types of nanosponges, including those made of metals [6], silicon [7], synthetic polymers [8,9], and polysaccharides [10]. These nanosponges, which are based on polysaccharides, use starch and cyclodextrins. Researchers are particularly interested in cyclodextrin-based cross-linked polymers, also known as “cyclodextrin nanosponges” (CDNS), for addressing significant bioavailability issues, such as inadequate solubility, slow dissolution rate, limited stability of some agents, as well as increasing their effectiveness and minimising unfavourable side effects [11]. In addition, unlike other nanosponges, CDNS have an advantage because cyclodextrin and its derivatives are biologically degradable and non-toxic. This could make the regulatory approval process for CDNS less complicated than it would be for other nanosponges [12].

Although CDNS are proving to be a helpful delivery nanocarrier for active pharmaceuticals and innovative uses of CDNS are being developed rapidly, a significant number of questions concerning their engineering methods and the effect that these methods have on the physicochemical properties of nanosponges have not been answered. Recent years have seen a surge in the number of ground-breaking publications in the field of nanosponges, all of which have come to the same conclusion: the method by which they are prepared is essential to the nanosponges’ ability to function effectively in the applications for which they were designed [13]. Applying a wide range of production techniques has accomplished the development of nanosponges with consistent nanosize and morphology. Conventional heating techniques, such as melt fusion and solvent evaporation, were initially reported to be utilised in synthesising CDNS. These techniques have traditionally been the most well liked and often applied. However, conventional procedures are unreliable because they yield non-uniform outcomes due to a sharp thermal gradient in the bulk arrangement and challenges with the preparative process. Due to the substantial amount of solvent needed for these conventional techniques and the reaction’s heat lability, mass scaling was challenging. Analysts noticed they needed to concentrate on how warming tactics affected the creation of nanosponges in this regard. They discovered that conventional methods rely on convection warming, which results in slow and uneven warming and cooling of the dissolvable medium. Other significant issues with traditional amalgamation techniques included a longer span and a low yield [14].

Microwave (MW)-assisted syntheses have been demonstrated to be superior to conventional warming methods because they can overcome the heterogeneity of heat dispersion in solutions [15]. Rapid reaction kinetics, effective processing, and favourable condensation effects brought on by peculiar, constrained “hotspot” heating has made MW-assisted heating very popular in many technical domains, including organic synthesis and the fabrication of inorganic materials [16]. This approach for synthesising porous nanocarriers [17] has the potential benefits of selectivity in phases, a narrow particle size range, and straightforward structural control, in addition to fast crystallisation. The consistent and accurate character of the heating system makes microwave-assisted responses more reproducible and deducible. In addition, the response temperature is precisely controlled and directed. As opposed to conventional thermal approaches, MW irradiation increases the temperature of the reaction mixture concurrently and independently of the container holding the mixture. This implies that the process occurs uniformly and concurrently throughout the reaction vessel. This impact affects total response adaptability because the same temperature profile may be given, regardless of vessel volume [18]. The reaction vessel’s temperature may increase during MW-assisted synthesis to the point where the utilised solvent begins to boil. This might accelerate the procedure 10–1,000 times faster. Hence, reactions take place swiftly, often just lasting a few seconds. The cycle may make it simple to produce nanocarriers because yields are significantly higher [19]. Additionally, materials made with microwave irradiation have different qualities and properties than those made with traditional heating methods.

The novelty of our work lies in the synthesis of nanosponges, which revolves around building a simple, low-cost, high-yield synthesis process, as well as optimising reaction conditions to increase yield and reduce preparation time. Optimisation of the boundaries of the interaction was carried out by incorporating the response surface technique, and the Box-Behnken design was adopted. The study adopts a non-conventional MW-assisted method to cross-link native β-cyclodextrin (β-CD) with a diphenyl carbonate (DPC) linker. The crystalline nature of CDNS prepared by the MW technique is responsible for enhanced drug loading, resulting in improved water-dissolvability of the model drug domperidone.

## 2. Results and Discussion

### 2.1. Phase Solubility Studies

**Figure 1:**
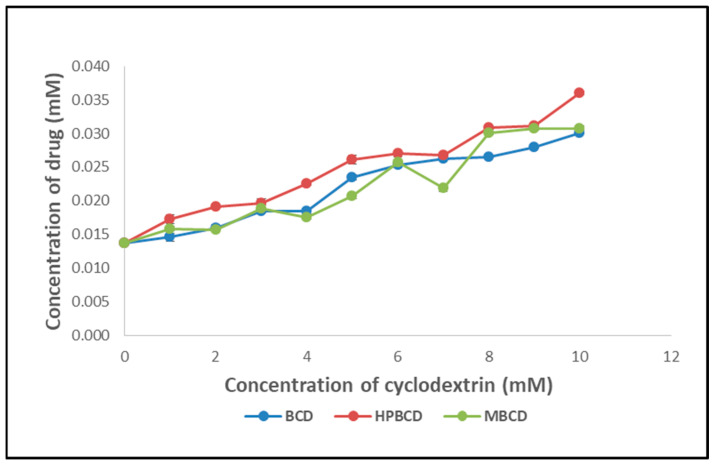
Phase solubility study of domperidone (DOM) with various cyclodextrins.

Based on the stability constants discovered during the phase solubility experiments (Figure 1) of DOM with various CDs, it was determined that hydroxypropyl βCD (HPβCD), which has a stability constant value of 143.14 M^−1^, improves the drug’s solubility. Hence, the initial step was creating nanosponges using HPβCD. Unfortunately, despite numerous trials, the obtained product remained slimy and could not be processed further. As a result, attention was drawn to the creation of CDNS. The stability constant for DOM with βCD was discovered to be 121.63 M^−1^. Since this value is likewise high enough, the following studies were carried out, utilising βCD. Its affordable price and easy availability inspired this decision, and ease of handling was also observed during CDNS synthesis.

To synthesise CDNS, the next critical step was selecting the linker. Through a review of the literature, we narrowed our selection to diphenyl carbonate (DPC) and carbonyldiimidazole. Diphenylcarbonate also has additional advantages, such as a low cost, ease of handling, and the ability to form products with desired characteristics [20]. Synthesis of nanosponges, employing carbonyldiimidazole as the linker, gave a slimy product that could not be treated any further. Hence, DPC was chosen as the linker for further experiments.

### 2.2. Optimisation

The trials were carried out in accordance with the plan, and the outcomes were discovered. Stat-Ease Design Expert^®^ V13.0 software was used to analyse the variances of the responses surfaces of the variables within the experimental domain. It was chosen because Box-Behnken provides a useful method for analysing quadratic response surfaces and modelling second-order polynomials [21]. The five-centric Box–Behnken design approach necessitates seventeen experiments. Variations in response were observed, depending on factor combinations, as shown in Table 1 below. The significant changes across all 17 batches show that the dependent variables rely heavily on the autonomous variables [22].

Based on multiple linear regression analysis, mathematical correlations were calculated for the variables summarised in Table 2. In this equation, MW power level (A), reaction time (B), and stirring speed (C) are quantitatively correlated with percentage yield (Y1) and particle size (Y2). Each coefficient of A, B, and C represents the effect of the variable on the response Y1 and Y2. A coefficient containing more than one component or a coefficient having higher-order terms illustrates interaction terms and quadratic relationships. Synergistic effects are indicated by a positive sign, while antagonistic effects are characterised by a negative sign. Both polynomial equations were determined to be statistically significant (*p* < 0.01) by ANOVA (Table 2 and Table 3), following the guidelines of the Design Expert^®^ software.

The F-value for the model is 68.29, which indicates its statistical significance. There is only a 0.01 percent chance of an F-value this large occurring due to noise. P-values for significant terms in the model are lower than 0.0500. The F-value of 0.7631 suggests that the lack-of-fit is insignificant compared to the pure error.

**Figure 2:**
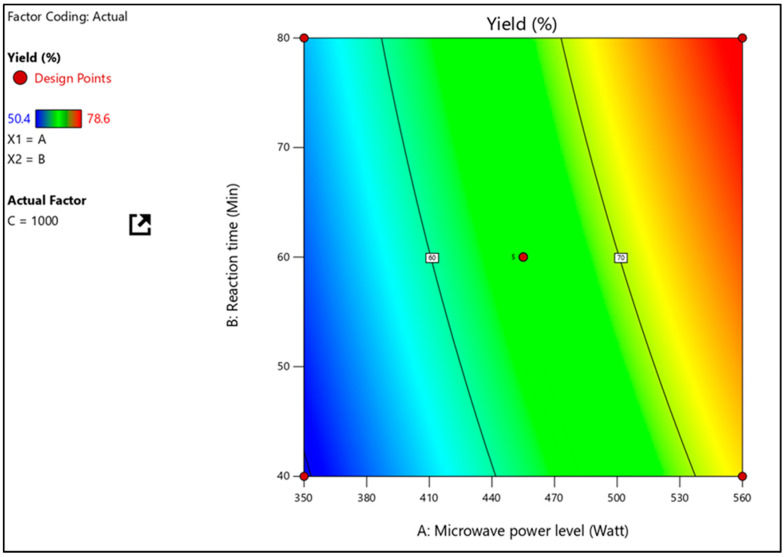
Response surface plot, presenting the interactions between the microwave power level and reaction time affecting the percentage yield at a constant stirring speed.

A F-value of 68.29 and a R^2^ value of 0.9887 were determined to be significant in the mathematical model for percentage yield (Y1). The independent terms A, B, and C, as well as the quadratic terms AB, AC, BC, and A^2^, significantly influence the yield, since the P values less than 0.05 indicate their statistical significance, as seen in Table 3. The conclusion to the equation is that A has a higher impact than B and C combined. It further investigated how independent parameters interacted with one another on practical yield using three-dimensional response surface plots. Figure 2 shows the interaction effect of A and B at a fixed C level on percent yield (Y1). With low levels of A, Y1 (percentage yield) increases from 52 to 57%. (MV power level). Similarly, Y1 increases from 74% to 80% when A levels are high.

In the model, the F-value is 471.01, indicating that the effect is substantial. Noise has a 0.01% chance of causing a F-value of this size. The lack of fit is insignificant compared to the pure error, according to its F-value of 1.41.

**Figure 3:**
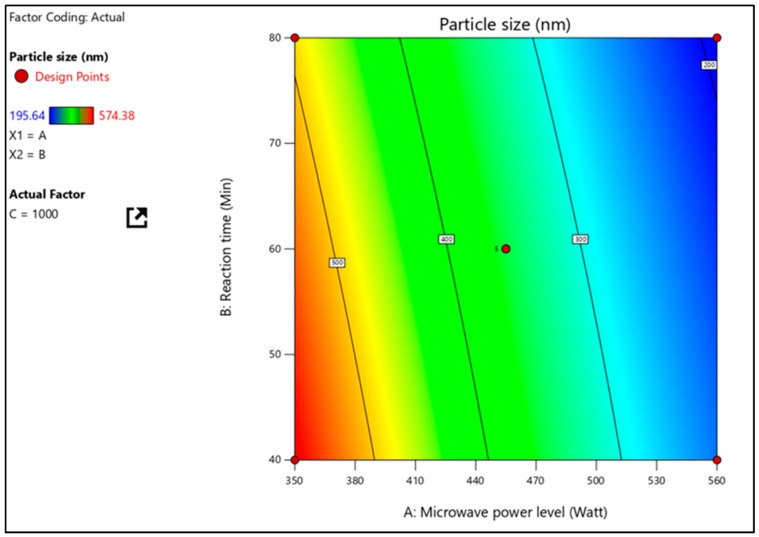
Particle size effects of interactions between microwave power level and stirring speed at constant reaction time are shown on a response surface plot.

According to Table 3, the particle size of nanosponges was between 194.72 and 560.46 nm. As indicated by the correlation coefficient (1.000) and the F-value (471.01), the factorial equation for particle size is statistically significant. The value of “Prob > F” must be less than 0.0001 for the model terms’ significance. As shown in Table 4, the model terms A, B, and C, as well as the quadratic terms AB, A^2^, and C^2^, are all significant. Factor A (MW power), which has the most significant impact, concludes that when power is raised, and smaller and smaller nanosponges are produced. A three-dimensional response surface plot was used to clarify further the effects of independent factors on particle size and their interactions using response surface plots. A three-dimensional response surface plot of the response Y2 (Figure 3) illustrates how independent variables interact to influence the response when one variable is held constant. At the same time, the other variables move within a specific range.

The Design Expert^®^ software was then used to perform numerical optimisation based on the desirability function. By adjusting the MV power level, reaction duration, and stirring speed within the study range, MW-NS was optimised to produce the smallest particle size with the highest yield. It was found that there are more than a hundred potential solutions, each with a unique combination of independent factors. Therefore, it was determined that one should select the solution with the highest attractiveness rating as the ideal processing condition. The final batch was stirred at 1000 rpm, while 560 W was applied for 80 min. In this instance, it was expected that 79.6% of the total yield and the formation of NSs with an average particle size of 194.72 nm would occur. The batch yield, however, was discovered to be 77.4% after scaling up, and the MW-NS had an average particle size of 195.68 ± 2.16 nm.

### 2.3. Synthesis of Cross-Linked βCD Nanosponges

The ratios of 1:2 and 1:4 (CD:DPC) were insufficient to create stable nanosponges, since polymer CD and the cross-linker DPC that were created with them decomposed during the Soxhlet extraction procedure. A 1:6 ratio between CD and the cross-linker DPC produced stable, powdered nanosponges [23]. A further trial was performed, in which no additional growth of the polymeric network was seen to evaluate the suitability of the 1:8 ratio. As a result, it was decided that 1:6 would be the final ratio for preparing nanosponges.

### 2.4. Evaluation of Drug-Loaded Nanosponges

#### 2.4.1. Fourier Transforms Infrared-Attenuated Total Reflectance Spectroscopy (FTIR-ATR) Analysis

**Figure 4:**
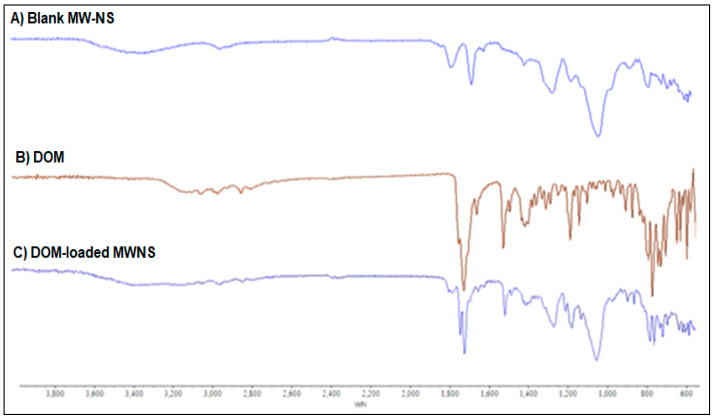
FTIR spectra of (**A**) blank CDNS, (**B**) DOM and (**C**) DOM-loaded CDNS.

For NSs made using the MV approach, the FTIR-ATR spectra of the NSs were measured in the frequency range of 600–4000 cm^−1^. The formation of CDNS was confirmed by the FTIR spectra of the NSs, which showed a clearly defined carbonate bond between two βCD molecules in the range of 1740–1780 cm^−1^ (Figure 4). The extra NS characteristic peak was also found at 2918 cm^−1^, 1418 cm^−1^, and 1026 cm^−1^, respectively, attributable to the primary alcohol’s C-O stretching vibration, C-H bending vibration, and C-H stretching vibration. The literature recommends the region between 1700 and 1800 cm^−1^ for assessing the crystallinity of CDNS [24]. Crystalline nanosponges’ C=O bonds displayed a slight peak shift of about +40 cm^−1^ at 1780.05 cm^−1^ [24]. In traditionally synthesised NSs, the typical peak of the carbonate bond between βCD units was seen at about 1740 cm^−1^, while in MW-manufactured NSs, it was shifted to a higher value (1780 cm^−1^). The primary alcohol group in βCD’s absence of the distinctive non-hydrogen-bonded O–H stretching at 3450 cm^−1^ proves that the cross-linking process has finished. At 1750 cm^−1^, carbonate NS has a prominent peak [25].

**Figure 5:**
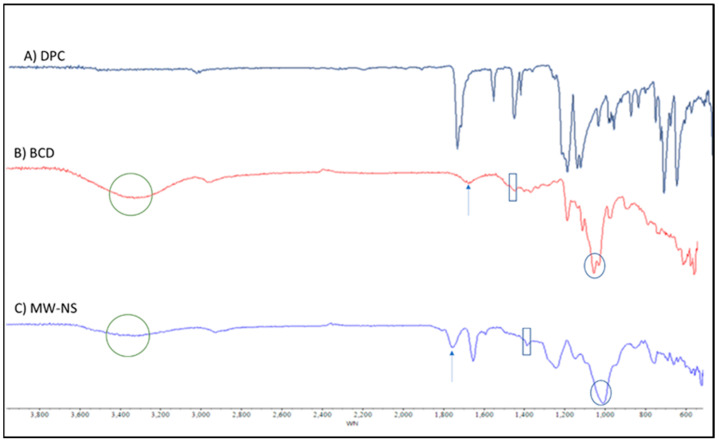
FTIR spectra of (**A**) DPC, (**B**) βCD, and (**C**) blank CDNS produced using the MW approach.

According to NS structural characterisations of the synthesised molecule, a carbonate linkage has been added to the parent CD unit’s main hydroxyl groups [26]. As a result, drug molecules might be integrated into CDs’ nanocavities, and cross-linking might allow for additional interactions between the guest molecules and other CD units. Cross-linking may also result in nanochannels forming inside the NS structure. This peculiar structural configuration could be why NS protects and solubilises better than its parent CD. FTIR-ATR spectra for NSs, made using MW techniques, were measured in the frequency range of 600–4000 cm^−1^. According to the FTIR analysis (Figure 4), plain DOM was indicated by distinctive peaks at 3070, 2933, 1718, and 1620 cm^−1^ caused by N-H stretching, asymmetric C-H stretching, and C=O stretching, respectively. Unloaded NS made using the MW approach exhibited unique peaks in the FTIR spectrum at about 1776 cm^−1^, which is both a characteristic of carbonate cross-linked NSs and a sign of its crystalline form. The primary alcohol group in CD typically exhibits the distinctive non-hydrogen-bonded O-H stretching at 3450 cm^−1^ as a sign that the cross-linking process has been completed (Figure 5). It was noted that the physical mixture contained all of the distinctive DOM peaks. Depending on the wave number, drug loading and encapsulation should cause the characteristic drug peaks to vanish, diminish, or change. The spectrum of the final DOM-loaded NS formulation had all prominent DOM peaks at extremely low intensities, confirming DOM encapsulation in the NSs (Figure 4). Considering the preceding, we can conclude that the CDNS did indeed encapsulate DOM.

#### 2.4.2. Determination of Particle Size and Zeta Potential

It was discovered that synthesised NSs had an average particle size of 194.72 ± 1.24 nm and a polydispersity index (PDI) of 0.218 ± 0.049. DOM was loaded into these NSs, and the resulting particles were 195.68 ± 2.16 nm in size and had a PDI of 0.257 ± 0.061 [27]. This demonstrates that the loaded and unloaded NSs have a colloidal size range with a homogeneous size distribution [28]. The zeta potential for the formulated CDNS and loaded CDNS was −10.1 ± 0.25 mV and −9.17 ± 0.43 mV, respectively. This could be considered sufficient to ensure that the individual particles maintain a healthy electrostatic distance from one another. It is possible that βCD’s carbonyl groups and free hydroxyl groups are what give it its negative charge. Thus, it is very likely that the generated CDNS will remain stable for the duration of the product’s shelf life.

#### 2.4.3. Drug-Loading and Drug-Encapsulation Efficiency

These NSs have drug loading and entrapment efficiencies of 42 ± 2.5% and 84 ± 4.2%, respectively. DOM may have been encapsulated in the lipophilic cavities of the NS structure, contributing to the high encapsulation efficiency of the formulation. When hydrogen atoms are present, DOM can also form hydrogen bonds. It can also do this when aromatic rings contact strongly with the protons of a βCD molecule through the van der Waals force of attraction.

#### 2.4.4. Differential Scanning Calorimetry (DSC)

**Figure 6:**
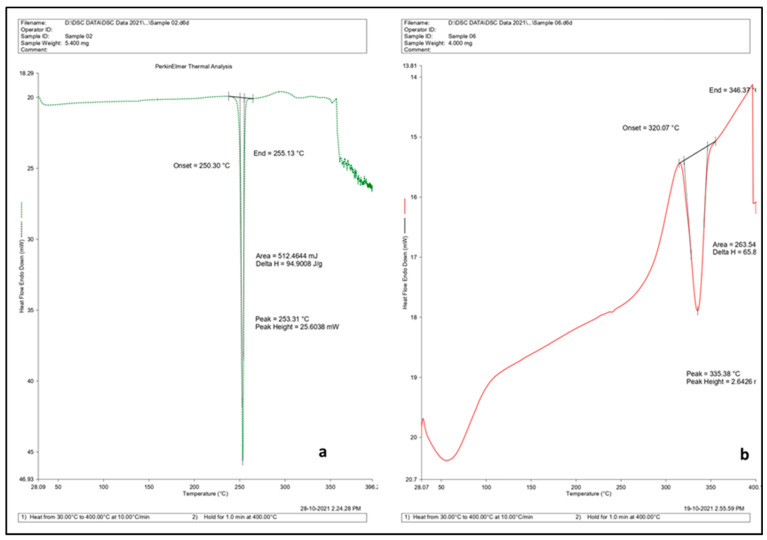
DSC thermogram of (**a**) DOM and (**b**) DOM-loaded CDNS.

The DSC thermogram of the drug, blank CDNS, and the drug-loaded CDNS is shown in Figure 6. DOM shows a characteristic peak at 253.31 °C. This peak reduced hinting toward the entrapment of the drug in the nanosponges formed and their subsequent amorphisation [29]. From DSC, it was found that prepared nanosponges degraded at 320 °C. The same results were reported by the published work of other researchers [14,30]. Trotta et al. reported the thermal degradation of CDs and substituted β-CDs. They found that, in inert atmosphere, they all decompose in one major step (252–400 °C), leaving a residue (Char), which is thermally quite stable, decomposing at a low rate at higher temperature [30]. In another study, the DSC thermogram of nanosponges had shown an exothermic peak at about 350–360 °C, which could be ascribed to degradation of the nanosponge. In case of nanosponges prepared by MV heating, an endothermic peak was observed at around 320 °C, which could be due to the presence of moisture in the crystal structure [14]. In addition, accelerated stability studies were also performed on optimized formulations. The results of stability studies showed no significant changes in physical parameters after storage on the 30th, 90th, and 180th days (Table S1).

#### 2.4.5. X-ray Powder Diffraction (XRPD)

**Figure 7:**
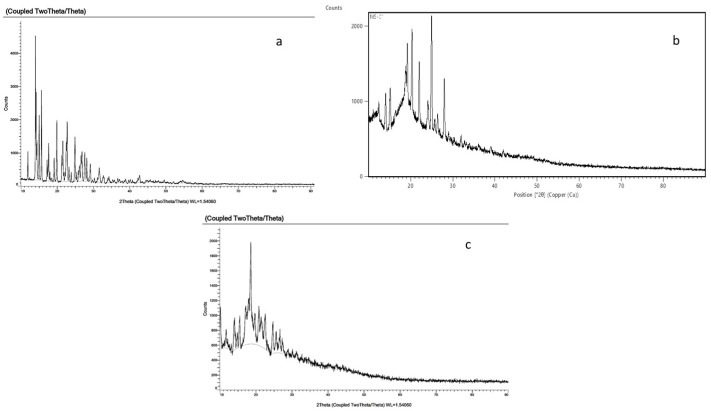
XRPD spectra of (**a**) DOM, (**b**) blank CDNS, and (**c**) DOM-loaded CDNS.

The diffraction pattern of the NS prepared by the MV-assisted synthetic route (Figure 7) showed changes in peaks and a slightly diffused pattern, hinting towards the crystalline/para-crystalline nature of the formed NS [31]. The XRD pattern of DOM confirmed its crystalline form. The diffraction pattern of the loaded CDNS prepared by the microwave method was diffused and different from that of DOM, ensuring the encapsulation and probable amorphisation of the drug [32].

#### 2.4.6. In Vitro Release Studies

**Figure 8:**
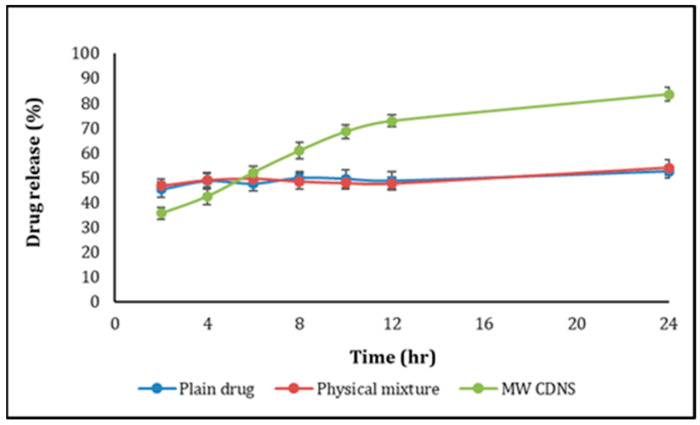
In vitro drug release profile of pure DOM, physical mixture of DOM/βCD, and DOM-loaded CDNS.

The USP dissolution tester II (Paddle method) was used to examine the release of DOM-loaded CDNS, pure DOM, and DOM-βCD/physical mixture. The paddles rotated at 50 rpm, and the temperature was 37 °C + 0.5 °C. Weighed and filled dialysis bags with DOM-loaded NS, having 15 mg DOM and 15 mg of pure DOM with or without βCD. There were three sets of release experiments, and the average results were shown as a function of time as a cumulative percent of medicine released. In addition, the release properties of drug-loaded NS and plain drug were compared using the difference factor (f1) and similarity factor (f2) [33]. Figure 8 depicts the drug release profile, and values for f1 and f2 were discovered to be 26 and 42, respectively. Regarding the release profiles, the profile of NSs loaded with the drug and that of the plain drug is entirely different. The amount of drug released from NSs prepared by the MW method increases significantly, as demonstrated by f1 and f2 values not falling within the prescribed ranges (range for f1 is < 15 and range for f2 is 50–100) [34,35].

#### 2.4.7. Morphological Evaluation

**Figure 9:**
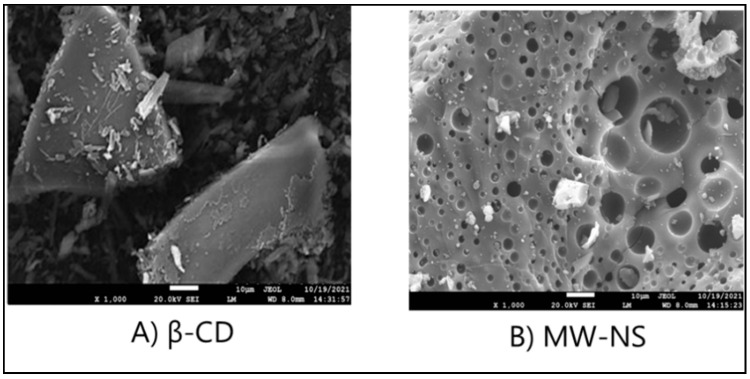
SEM scan of (**A**) βCD and (**B**) CDNS.

SEM studies were conducted to determine particle morphology. SEM topography images of β-CD and nanosponges after drug loading are shown in Figure 9 at magnifications of 30,000×. The SEM image of β-CD showcases its crystalline nature. When β-CD molecules are linked with a linker to create nanosponges, the distinctive sponge-like structure of nanosponges is observed in samples. A recent study found that nanosponges retained their sponge-like morphology even after lyophilisation. Because our samples are porous, drugs may be loaded and delivered more efficiently [36].

## 3. Materials and Methods

### 3.1. Materials

DOM was provided as a gift sample from Ankur Drugs and Pharma Ltd. (Baddi, India). Cyclodextrins (βCD, HPβCD, and MβCD) were generously donated by Roquette India Private Ltd. (Mumbai, India). DPC was purchased from Sigma Aldrich (Mumbai, India). Analytical-grade chemicals and reagents were used for the rest of the experiment. The experiments made use of Millipore’s Milli-Q water.

### 3.2. Phase Solubility Studies

As described by Higuchi and Connors, phase solubility studies were conducted between DOM and different cyclodextrins to determine their degree of complexation [37]. In a series of clear vials, extra drug crystals (50 mg) were added to 10 mL of water that contained different amounts of βCD (0–10 mM), HPβCD (0–10 mM), and MβCD (0–10 mM). The suspensions were then agitated using a vibratory shaker for 48 h at room temperature. Afterwards, the suspensions were shaken at ambient temperature for 48 h using a vibratory shaker. Finally, the dispersions were filtered after equilibration using Whatman filter paper (No. 40). To determine the DOM concentration from filtered samples, UV analysis was conducted and compared to a blank. At 287 nm, the exact quantities of βCD, HPβCD, and MβCD did not exhibit any discernible absorption. Therefore, each experiment was performed in triplicate. The phase solubility diagrams were created by plotting the DOM concentrations against the corresponding CD concentrations. Based on their incline and intercept values, we calculated the binding constants using the following equation:

(1) $$K_{1:1}=\frac{Slope}{S_{0}\left(1-slope\right)}$$

where *S*_0_ signifies the drug’s intrinsic solubility (i.e., its solubility in aqueous solutions in the absence of CD), and the slope denotes the inclination of the linear drug-CD solubility curve (i.e., AL-type).

### 3.3. Optimisation

Based on preliminary findings, a Box-Behnken experimental design was employed to examine the effects of three different levels of MV power (A), reaction duration (B), and stirring speed (C) on the quantity of polymerisation. In addition, particle size and practical yield were considered in this design. Table 5 and Table 6 display the list of variables that are both independent and dependent [38,39].

The experiments were carried out, as specified by the design, and the outcomes were collected. To obtain an ANOVA and to examine the response surfaces of the variables in the experimental domain, Stat-Ease Design Expert^®^ software V13.0 was utilised.

### 3.4. Synthesis of Cross-Linked βCD NSs

For MV reactions, a 2450 MHz MV system (Raga Tech, Pune, India) was employed. It has an infrared camera and a fibre optic temperature probe. Magnetic stirrers were used to perform the stirring. Maintaining and monitoring the reaction environment, which is essential to the creation of NSs, is made much easier by integrated systems. In a 250 mL flask containing 100 mL of a suitable solvent (dimethyl formamide), the polymer (βCD) and cross-linker (DPC) were mixed in different molar ratios, ranging from 1:2 to 1:6 before MW irradiation [40]. Then, the reaction was terminated, and the solvent was removed by distillation. After the solvent had been entirely eliminated, the product was rinsed with water before being purified with ethanol using a Soxhlet extraction process for around 4 h. The final white powdery product was ground in a mortar and stored at 25 °C after being dried overnight in an oven at 60 °C [38].

### 3.5. Loading of the Drug into NSs

A modified loading method that involves solvent evaporation and freeze-drying was used to load DOM into the nanocavities of synthesised NSs [41,42]. In a nutshell, DOM-loaded NSs were produced using optimal formulations of NSs and drug powder (pure DOM) in an equimolar ratio. A precisely weighted amount of the CDNS was homogenised for 10 min in 30 mL of water. Following this, the homogenised liquid was sonicated for 10 min with the same amount of medication (DOM) dissolved. As a result of using a magnetic stirrer, this mixture was stirred continuously for 24 h. The uncomplexed drug was collected from the aqueous suspension by centrifugation at 2000 rpm for ten minutes. DOM-loaded nanosponges were separated by drying the supernatant in a lyophiliser (Bio gene, India). The drug-loaded lyophilised NS was kept at room temperature in a desiccator before being used again. The following calculations would be used to compute the drug loading and% entrapment efficiency:

(2) $$Loading\;capacity\;\left(\%\right)=\frac{Amount\;of\;drug\;in\;the\;CDNS}{Amount\;of\;CDNS\;from\;which\;drug\;is\;\;extracted}\times 100$$

(3) $$Entrapment\;efficiency\;\left(\%\right)=\frac{Amount\;of\;drug\;in\;the\;CDNS}{The\;initial\;amount\;of\;drug\;added}\times 100$$

### 3.6. Evaluation of Drug-Loaded Nanosponges

#### 3.6.1. FTIR-ATR

The frequency range 4000–600 cm^−1^ was used to measure the FTIR-ATR spectra of βCD, DPC, CDNS, DOM, and drug-loaded NSs, produced by the MV-assisted technique, using the FTIR-3000B (Analytical Technologies Ltd., Mumbai, India). An excellent signal-to-noise ratio and outstanding repeatability were ensured by recording each spectrum at a resolution of 4 cm^−1^ in a dry environment to prevent the introduction of contaminating components. There was no mathematical correction (such as smoothing).

#### 3.6.2. Determination of Particle Size and Zeta Potential

The mean particle size, PDI, and zeta potential of pure DOM and the DOM-loaded CDNS were measured using an industry-standard Malvern^®^ Zetasizer Nano ZS 90 (Malvern^®^ Instruments Limited, Worcestershire, UK). A triplicate measurement of each sample was conducted at 25 °C using a scattering angle of 90° after diluting it (1:200) in deionised water [43].

#### 3.6.3. Determination of Drug Loading and Encapsulation Efficiency

DOM-loaded nanosponge formulations were rinsed thoroughly with dichloromethane to remove any free drug. Drying, triturating with methanol, and sonicating them for 15 min released the encapsulated drug. After filtering the solutions, they were spectrophotometrically analysed at 287 nm [44,45].

#### 3.6.4. DSC

DSC was carried out, employing a DSC 7020 differential scanning calorimeter (Hitachi, Japan). Calibration was performed with indium for melting point and heat of fusion. The heating rate was set at 10 °C/min in the 35–300 °C temperature range. An empty aluminium sample pan served as a reference standard. In triplicate, 5 mg samples were analysed under nitrogen purge. This was performed to determine whether the drug had been incorporated into NS.

#### 3.6.5. XRPD

To understand the differential crystallinity behaviour of plain NS and the loaded one, we carried out a detailed XRPD study using a Bruker diffractometer (D8 Advance, Coventry, UK). Samples were scanned using a copper line as a radiation source at a scanning rate of 3°min^−1^ over a 2θ range between 5–90° angle at a 40 kV voltage and 40 mA current.

#### 3.6.6. In Vitro Drug Release Studies

For the assessment of the drug release from the CDNS, 0.1 N HCl was used for the first two hours, and phosphate buffer pH 6.8 was used for the remaining 22 h. A physical mixture of the DOM and βCD (1:1), pure DOM, and the required quantity of DOM-loaded NSs were all added to a 1.5-inch dialysis membrane (MWCO 12,000 Da). Before adding 1 mL of 0.1 N HCl, the dialysis membrane was presoaked in water to help distinguish between its two layers. One end of the membrane was then tied with thread. Loaded dialysis membranes were put in a USP type II dissolution apparatus filled with a 500 mL dissolution medium. At 2, 4, 6, 8, 10, 12 and 24 h, 5 mL aliquots were removed, and new dissolution media were added each time. At 287 nm, filtered samples were examined using spectrophotometry [46]. The investigation was repeated three times to guarantee its reliability.

#### 3.6.7. Morphological Investigation

Field emission scanning electron microscope (FE-SEM, JSM-7610F Plus, JEOL) was used to determine the surface morphology of the particles. Using a double-sided carbon adhesive tape, the nanosponges were frivolously scattered on the tape. Those were then wedged on gold-coated aluminium stubs of 300 Å to reduce the charging effects, and photomicrographs were taken at an accelerating voltage of 20 kV.

## 4. Conclusions

The current work aimed to synthesise CDNS for DOM, a BCS class II medication, utilising a greener, MW-based method to increase the molecule’s water solubility and speed up the manufacturing process, increase yield, and use fewer organic solvents. Several CDs and linkers were tested to find the most remarkable potential combination. Based on stability constants and product quality, βCD was determined to be the most acceptable CD for the intended usage. The Box Behnken-based technique successfully streamed the MW-based process by optimising the various process parameters. The formation of the blank NSs due to the reaction between the CD and the linker was confirmed by FTIR. The prepared NSs showed an entrapment efficiency of 84 ± 4.2%. A significant improvement in the in vitro drug release was observed compared to the pure drug. The MW-based strategy accelerated the process, and the yield was appreciably higher than with more traditional techniques, such as solvent evaporation and melting.

Consequently, we could unequivocally demonstrate that this might be an effective strategy for improving the solubility of BCS class II compounds, which might then improve their pharmacokinetics. Further work needs to be performed to scan various drugs, as well as to streamline the drug selection and entrapment process. The method explored was greener, as compared to the conventional techniques, but it produced toxic by-products, such as phenol, as well as the presence of unreacted cross-linking agents. The removal of both of these required the use of organic solvents, such as ethanol or acetone, and it also required a lengthy Soxhlet extraction. Thus, more work must be performed to shorten the time and effort necessary for the purification process. Although the raw materials used in the synthesis of the NSs enjoy the GRAS status, extensive work was also performed to purify the prepared NSs, and acute and long-term toxicity studies need to be performed to comment on the safety of these nanocarriers conclusively. The final confirmation of the hypothesis can only be obtained by conducting the preclinical studies, the results of which, if found suitable, could translate to the clinical stage.

## Figures and Tables

**Table 1:** Summary of Box–Behnken plan and measured responses.

Run	Factors			Responses	
	A: Microwave Power Level (Watt)	B: Reaction Time (min)	C: Stirring Speed (rpm)	Yield (%)	Particle Size (nm)
1	560	60	1500	77.7	203.14
2	560	40	1000	74.4	232.87
3	350	80	1000	57.5	482.31
4	455	60	1000	66.2	331.30
5	455	80	1500	68.2	294.29
6	455	60	1000	64.9	321.28
7	455	60	1000	65.3	322.17
8	350	60	1500	55.6	502.83
9	455	40	500	61.7	386.69
10	455	60	1000	65.8	317.56
11	560	60	500	75.2	211.93
12	350	40	1000	52.7	563.46
13	455	40	1500	62.2	360.16
14	455	60	1000	65.2	324.45
15	455	80	500	67.4	309.42
16	560	80	1000	79.6	192.72
17	350	60	500	54.1	531.37

**Table 2:** Regression equations for the measured responses, Y1 and Y2.

Response	Code	Equation
Yield (%)	Y1	65.51 + 10.8623 * A + 2.715 * B + 0.6623 * C + 0.1 * AB + 0.277 * AC + 0.04 * BC + 0.6777 * A^2^ + −0.1475 * B^2^ + −0.5225 * C^2^
Particle size (nm)	Y2	328.763 − 155.168 * A − 31.0537 * B − 8.6277 * C + 9.1 * AB + 3.1824 * AC + 3.5977 * BC + 33.2518 * A^2^ + 6.5765 * B^2^ + 2.049 * C^2^

**Table 3:** The ANOVA outcomes of the quadratic model for the measured outcome yield (%).

Source	Sum of Squares	df	Mean Square	F-Value	*p*-Value	
Model	1178.95	9	130.99	68.29	<0.0001 *	Significant
A-Microwave power level	1083.45	1	1083.45	564.82	<0.0001 *	
B-Reaction time	85.15	1	85.15	44.39	0.0003 *	
C-Stirring speed	4.2	1	4.2	2.19	0.0018 *	
AB	0.4225	1	0.4225	0.2203	0.0065 *	
AC	0.81	1	0.81	0.4223	0.0053 *	
BC	1.44	1	1.44	0.7507	0.0041 *	
A^2^	1.11	1	1.11	0.5765	0.0047 *	
B^2^	0.4112	1	0.4112	0.2144	0.6574	
C^2^	2.14	1	2.14	1.11	0.3262	
Residual	13.43	7	1.92			
Lack of Fit	4.89	3	1.63	0.7631	0.5709	Not significant
Pure Error	8.54	4	2.13			
Cor Total	1192.38	16				
R^2^						0.9887
Adjusted R^2^						0.9743
Predicted R^2^						0.9232

* *p* < 0.01—statistically significant.

**Table 4:** The ANOVA outcomes of the quadratic model for the measured response Y2.

Source	Sum of Squares	df	Mean Square	F-Value	*p*-Value	
Model	2.17 × 10^5^	9	24063.23	471.01	<0.0001 *	Significant
A-Microwave power level	2.04 × 10^5^	1	2.04E+05	3984.25	<0.0001 *	
B-Reaction time	8988.05	1	8988.05	175.93	<0.0001 *	
C-Stirring speed	705	1	705	13.8	0.0075 *	
AB	379.28	1	379.28	7.42	0.0296 *	
AC	21.53	1	21.53	0.4214	0.5369	
BC	80.1	1	80.1	1.57	0.2507	
A^2^	2227.8	1	2227.8	43.61	0.0003 *	
B^2^	14.22	1	14.22	0.2783	0.6141	
C^2^	736.45	1	736.45	14.42	0.0067 *	
Residual	357.62	7	51.09			
Lack of Fit	183.59	3	61.2	1.41	0.3637	Not significant
Pure Error	174.03	4	43.51			
Cor Total	2.17 × 10^5^	16				
R^2^						0.9984
Adjusted R^2^						0.9962
Predicted R^2^						0.9852

* *p* < 0.05—statistically significant.

**Table 5:** List of independent factors selected in the Box–Behnken design to optimise the MV-assisted synthesis of NS.

Name of the Independent Variable	Code	Unit	Levels		
			Low	Medium	High
MW power	A	Watt	350	455	560
Reaction time	B	Min	40	60	80
Stirring speed	C	rpm	500	1000	1500

**Table 6:** List of dependent factors selected in the Box–Behnken design to optimise the MV-assisted synthesis of NS.

Name of the Dependent Variable	Unit	Goal
Yield	%	Maximise
Particle size	nm	Minimise

## Data Availability

All of the relevant data are provided in the form of regular Figures and Tables.

## Supplementary Materials

The following supporting information can be downloaded at: https://www.mdpi.com/article/10.3390/ph16040567/s1, Table S1. Physical parameters of DOM loaded into MW-NS subjected to stability studies. The stability procedures and results are also included under supplementary materals.

## References

[1] Amidon G.L., Lennernäs H., Shah V.P., Crison J.R. (1995). A theoretical basis for a biopharmaceutic drug classification: The correlation of in vitro drug product dissolution and in vivo bioavailability. Pharm. Res..

[2] Patel D.M., Patel S.P., Patel C.N. (2014). Formulation and evaluation of fast dissolving tablet containing domperidone ternary solid dispersion. Int. J. Pharm. Investig..

[3] Subramanian S., Anandam S., Kannan K., Rajappan M. (2012). Nanosponges: A novel class of drug delivery system--review. J. Pharm. Pharm. Sci..

[4] Ahmed R.Z., Patil G., Zaheer Z. (2013). Nanosponges—A completely new nano-horizon: Pharmaceutical applications and recent advances. Drug Dev. Ind. Pharm..

[5] Lembo D., Trotta F., Cavalli R. (2018). Cyclodextrin-based nanosponges as vehicles for antiviral drugs: Challenges and perspectives. Nanomedicine.

[6] Pandey P., Purohit D., Dureja H. (2018). Nanosponges—A promising novel drug delivery system. Rec. Pat. Nanotechnol..

[7] Iravani S., Varma R.S. (2022). Nanosponges for drug delivery and cancer therapy: Recent advances. Nanomaterials.

[8] Tiwari K., Bhattacharya S. (2022). The ascension of nanosponges as a drug delivery carrier: Preparation, characterization, and applications. J. Mater. Sci: Mater. Med..

[9] Ahmed M.M., Fatima F., Anwer M.K., Ansari M.J., Das S.S., Alshahrani S.M. (2020). Development and characterization of ethyl cellulose nanosponges for sustained release of brigatinib for the treatment of non-small cell lung cancer. J. Polym. Eng..

[10] Atchaya J., Girigoswami A., Girigoswami K. (2022). Versatile applications of nanosponges in biomedical field: A glimpse on SARS-CoV-2 management. BioNanoScience.

[11] Krabicová I., Appleton S.L., Tannous M., Hoti G., Caldera F., Rubin Pedrazzo A., Cecone C., Cavalli R., Trotta F. (2020). History of cyclodextrin nanosponges. Polymers.

[12] Shende P., Kulkarni Y.A., Gaud R.S., Deshmukh K., Cavalli R., Trotta F., Caldera F. (2015). Acute and repeated dose toxicity studies of different β-cyclodextrin-based nanosponge formulations. J. Pharm. Sci..

[13] Singh D., Soni G.C., Prajapati S.K. (2016). Recent advances in nanosponges as drug delivery system: A review. Eur. J. Pharm. Med. Res..

[14] Singireddy A., Rani Pedireddi S., Nimmagadda S., Subramanian S. (2016). Beneficial effects of microwave assisted heating versus conventional heating in synthesis of cyclodextrin based nanosponges. Mater. Today Proc..

[15] Sharma N., Sharma U.K., Van der Eycken E.V. (2018). Microwave-assisted organic synthesis: Overview of recent applications. Green Techniques for Organic Synthesis and Medicinal Chemistry.

[16] Nadagouda M.N., Speth T.F., Varma R.S. (2011). Microwave-assisted green synthesis of silver nanostructures. Acc. Chem. Res..

[17] Jhung S.H., Lee J.H., Chang J.S. (2005). Microwave synthesis of a nanoporous hybrid material, chromium trimesate. Bull. Kor. Chem. Soc..

[18] Leonelli C., Lojkowski W. (2007). Main development directions in the application of microwave irradiation to the synthesis of nanopowders. Chem. Today.

[19] Dahal N., García S., Zhou J., Humphrey S.M. (2012). Beneficial effects of microwave-assisted heating *versus* conventional heating in noble metal nanoparticle synthesis. ACS Nano.

[20] Pushpalatha R., Selvamuthukumar S., Kilimozhi D. (2018). Hierarchy analysis of different cross-linkers used for the preparation of cross-linked cyclodextrin as drug nanocarriers. Chem. Engl. Commun..

[21] Zhang Q.Z., Tian X.F., Du G.L., Pan Q., Wang Y., Zhang X.L. (2013). Microwave-assisted synthesis of isopropyl β-(3, 4-dihydroxyphenyl)-α-hydroxypropanoate. J. Chem..

[22] Singireddy A., Pedireddi S.R., Subramanian S. (2019). Optimization of reaction parameters for synthesis of Cyclodextrin nanosponges in controlled nanoscopic size dimensions. J. Polym. Res..

[23] Castiglione F., Crupi V., Majolino D., Mele A., Rossi B., Trotta F., Venuti V. (2012). Effect of cross-linking properties on the vibrational dynamics of cyclodextrins-based polymers: An experimental–numerical study. J. Phys. Chem. B.

[24] Swaminathan S., Vavia P.R., Trotta F., Cavalli R., Tumbiolo S., Bertinetti L., Coluccia S. (2013). Structural evidence of differential forms of nanosponges of beta-cyclodextrin and its effect on solubilization of a model drug. J. Incl. Phenom. Macrocycl. Chem..

[25] Swaminathan S., Trotta F., Trotta F., Mele A. (2019). Cyclodextrin nanosponges. Nanosponges.

[26] Swaminathan S. (2006). Studies on Novel Dosage Forms. Masters Thesis.

[27] Deshpande A., Patel P. (2014). Preparation and evaluation of cyclodextrin based atorvastatin nanosponges. Am. J. PharmTech Res..

[28] Lembo D., Swaminathan S., Donalisio M., Civra A., Pastero L., Aquilano D., Vavia P., Trotta F., Cavalli R. (2013). Encapsulation of acyclovir in new carboxylated cyclodextrin-based nanosponges improves the agent’s antiviral efficacy. Int. J. Pharm..

[29] Khan S.A., Azam W., Ashames A., Fahelelbom K.M., Ullah K., Mannan A., Murtaza G. (2020). β-cyclodextrin-based (IA-co-AMPS) semi-IPNs as smart biomaterials for oral delivery of hydrophilic drugs: Synthesis, characterization, in-vitro and in-vivo evaluation. J. Drug Deliv. Sci. Technol..

[30] Trotta F., Zanetti M., Camino G. (2000). Thermal degradation of cyclodextrins. Polym. Degrad. Stab..

[31] Taleb S.A., Motasim Y., GabAllah M., Asfour M.H. (2022). Quercitrin loaded cyclodextrin based nanosponge as a promising approach for management of lung cancer and COVID-19. J. Drug Deliv. Sci. Technol..

[32] Caldera F., Tannous M., Cavalli R., Zanetti M., Trotta F. (2017). Evolution of cyclodextrin nanosponges. Int. J. Pharm..

[33] Kumari A., Jain A., Hurkat P., Verma A., Jain S.K. (2016). Microsponges: A pioneering tool for biomedical applications. Crit. Rev. Ther. Drug Carr. Sys..

[34] Shoaib Q.-u.-a., Abbas N., Irfan M., Hussain A., Arshad M.S., Hussain S.Z., Latif S., Bukhari N.I. (2018). Development and evaluation of scaffold-based nanosponge formulation for controlled drug delivery of naproxen and ibuprofen. Trop. J. Pharm. Res..

[35] Martin R., Sánchez I., Cao R., Rieumont J. (2006). Solubility and kinetic release studies of naproxen and ibuprofen in soluble epichlorohydrin-β-cyclodextrin polymer. Supramol. Chem..

[36] Singh V., Xu J., Wu L., Liu B., Guo T., Guo Z., York P., Gref R., Zhang J. (2017). Ordered and disordered cyclodextrin nanosponges with diverse physicochemical properties. RSC Adv..

[37] Higuchi T., Connors K.A. (1965). Phase solubility techniques. Adv. Anal. Chem. Inst..

[38] Anandam S., Selvamuthukumar S. (2014). Optimization of microwave-assisted synthesis of cyclodextrin nanosponges using response surface methodology. J. Porous Mater..

[39] Pongsumpun P., Iwamoto S., Siripatrawan U. (2020). Response surface methodology for optimization of cinnamon essential oil nanoemulsion with improved stability and antifungal activity. Ultrason. Sonochem..

[40] Zainuddin R., Zaheer Z., Sangshetti J.N., Momin M. (2017). Enhancement of oral bioavailability of anti-HIV drug rilpivirine HCl through nanosponge formulation. Drug Dev. Ind. Pharm..

[41] Omar S.M., Ibrahim F., Ismail A. (2020). Formulation and evaluation of cyclodextrin-based nanosponges of griseofulvin as pediatric oral liquid dosage form for enhancing bioavailability and masking bitter taste. Saudi Pharm. J..

[42] Zidan M.F., Ibrahim H.M., Afouna M.I., Ibrahim E.A. (2018). In vitro and in vivo evaluation of cyclodextrin-based nanosponges for enhancing oral bioavailability of atorvastatin calcium. Drug Dev. Ind. Pharm..

[43] Yaşayan G., Şatıroğlu Sert B., Tatar E., Küçükgüzel İ. (2020). Fabrication and characterisation studies of cyclodextrin-based nanosponges for sulfamethoxazole delivery. J. Incl. Phenom. Macrocyc. Chem..

[44] Ansari K.A., Vavia P.R., Trotta F., Cavalli R. (2011). Cyclodextrin-based nanosponges for delivery of resveratrol: In vitro characterisation, stability, cytotoxicity and permeation study. AAPS PharmSciTech.

[45] Torne S.J., Ansari K.A., Vavia P.R., Trotta F., Cavalli R. (2010). Enhanced oral paclitaxel bioavailability after administration of paclitaxel-loaded nanosponges. Drug Deliv..

[46] Osmani R.A., Thirumaleshwar S., Bhosale R.R., Kulkarni P.K. (2014). Nanosponges: The spanking accession in drug delivery-an updated comprehensive review. Der. Pharm. Lett..

