# Supplementary material for: Optimisation of a Greener-Approach for the Synthesis of Cyclodextrin-Based Nanosponges for the Solubility Enhancement of Domperidone, a BCS Class II Drug
Source: Pharmaceuticals (Basel). 2023 Apr 10;16(4):567. doi: 10.3390/ph16040567 (PMC10144918; doi:10.3390/ph16040567)
Supplement: Supplementary file 1 [file pharmaceuticals-16-00567-s001.zip › pharmaceuticals-2276925-supplementary.pdf]

Supplementary Materials

Stability studies

As suggested by the ICH Q1A (R2) guidelines, the optimized DOM-loaded into MW-NSs were tested for six months to see how stable they were in two different conditions. Based on these rules, temperatures and levels of humidity were chosen as per zones III and IV. The formulations were stored in stability chambers maintained at 40ºC/75% RH and 30ºC/65% RH and evaluated for their appearance, in vitro disintegration time, in vitro drug release and content of active ingredient with freshly prepared formulations at 30, 90 and 180 days of storage period.

Results

The physical parameters after the 30th, 90th, and 180th day are mentioned in Table S1. All the physical parameters were within the acceptable limits which showed that formulation was stable over 180 days.

Table S1. Physical parameters of DOM loaded into MW-NS subjected to stability studies.

Physical Parameter	Storage conditions	0	30	90	180
Appearance	40ºC/75% RH	+++	+++	+++	+++
	30ºC/65% RH	+++	+++	+++	+++
Disintegration Time(s)	40ºC/75% RH	25	25	23	24
	30ºC/65% RH	25	24	25	25
Drug content (%)	40ºC/75% RH	99.57	99.65	99.48	99.61
	30ºC/65% RH	99.57	99.42	99.36	99.17
In vitro drug release of DOM at the end of 24 hrs	40ºC/75% RH	83.00	83.64	83.21	83.49
	30ºC/65% RH	83.00	82.94	83.62	83.71

+++ Excellent, ++ Good, + Satisfactory, and – Poor
